# Comparison between a new thyroglobulin assay with the well‐established Beckman Access immunoassay: A preliminary report

**DOI:** 10.1002/jcla.23589

**Published:** 2020-09-20

**Authors:** Michele Cennamo, Evelina La Civita, Anna Curci, Antonietta Liotti, Umberto Braschi, Daniela Terracciano

**Affiliations:** ^1^ Department of Translational Medical Sciences University of Naples “Federico II” Naples Italy

**Keywords:** fine‐needle washout, immunoassays, rheumatoid factor, thyroglobulin, thyroglobulin auto‐antibodies

## Abstract

**Objectives:**

Measurement of serum thyroglobulin (Tg) plays a key role in the post‐thyroidectomy management of differentiated thyroid carcinoma (DTC). In this context, the performance of new‐generation thyroglobulin assay has clinical implications in the follow‐up of DTC patients. Aim of this study was to compare the new highly sensitive Liaison Tg II (Tg‐L) with the well‐established Tg Access assay (Tg‐A).

**Materials and methods:**

A total of 91 residual serum samples (23 positive and 68 negatives for Tg auto‐antibodies) were tested by the Beckman Access and Diasorin Liaison assays. Study samples were from 21 patients with pathologically proven DTC and control samples from 70 (16 patients with benign thyroid disease and 54 apparently healthy subjects).

**Results:**

Our results showed that Tg‐L was highly correlated with Tg‐A for both values ranging between 0.2 and 50 ng/mL (Pearson's *r* = 0.933 [95%CI 0.894‐0.958], *P* < .001) and higher than 50 ng/mL (Pearson's *r* = 0.849 [95%CI 0.609‐0.946], *P* < .001). For Tg values lower than 0.2 ng/mL, the overall concordance rate was 92%. Moreover, we tested 7 fine‐needle aspiration washout fluids (FNA), showing an overall concordance rate in discriminating negative and positive of 100%. Finally, we found no interference by Tg auto‐antibodies (TgAbs) for both Tg‐L and Tg‐A. Conversely, rheumatoid factor (RF) interferes with Tg‐A, but not with Tg‐L in one patient with no relapsing thyroid carcinoma.

**Conclusions:**

Liaison Tg II demonstrated a good correlation with Access Tg assay both for sera and FNAs. Further studies on larger population are needed to evaluate Tg‐L clinical impact on DTC patient's follow‐up.

## 1 INTRODUCTION

Serum Tg is an organ‐specific biomarker mirroring the thyroid volume.^1^ It has been estimated that 1ng/mL of Tg is equivalent to 1g of thyroid mass.^2^ Following total thyroidectomy serum, Tg levels are expected to be undetectable. On this basis, postoperative circulating Tg levels are used as a tumor marker to assess completely surgical resection and recurrence in the follow‐up of well‐differentiated thyroid cancer (DTC).^3^ After surgery, a Tg value >1‐2 ng/mL may suggest persistence of disease.^4^ Conversely, Tg levels lower than 1‐2 ng/mL after TSH stimulation define as negative for biochemical recurrence.^3^ Interestingly, unstimulated highly sensitive Tg Assays (hsTg) have a very good functional sensitivity (0.1‐0.2 ng/mL) and allow to avoid TSH stimulation.^5^, ^6^, ^7^ However, postoperative Tg predictive value can be affected by several factors such as TSH concentration at the moment of Tg measurement, time since total thyroidectomy, and functional sensitivity of the Tg assay.^8^, ^9^ Moreover, preoperative Tg is not recommended as screening since a lot of benign disease (Grave's disease, thyroiditis, or toxic nodules) may lead to higher serum Tg levels.^1^

Recently, the addition of Tg concentration of fine‐needle washouts (FNA‐Tg) of the suspicious lymph node to fine‐needle aspiration cytology (FNAC) showed improved negative predictive value (NPV) in the prediction of lymph node metastases (LNM).^10^, ^11^, ^12^

Collectively, Tg is a key tool in the clinical management of DTC patient and new‐generation thyroglobulin assay performance has implications for follow‐up of DTC.^13^, ^14^, ^15^ It is worthy of attention that anti‐immunoglobulin antibodies such as TgAbs, rheumatoid factor (RF), and anti‐human animal antibodies (HAMA) are able to interfere with immunometric Tg assays.^16^, ^17^ However, new developed tests have been formulated to minimize these interferences.^18^

Recently, a new automated highly sensitive Tg assay has become available: Liaison Tg II assay (Diasorin, Saluggia, Italy). At present, this assay has not been compared with other assays. Therefore, in this study we compared Liaison Tg II assay with the well‐established Access Tg immunoassay in a group of selected patients.

## 2 MATERIALS AND METHODS

### 2.1 Serum samples

A total of 91 residual serum samples were selected from our routine workload at University of Naples Federico II Hospital between September 2019 and December 2019. These subjects comprised 35 men and 56 women, and their mean age was 48.2 ± 17.0 years. Abnormal samples, such as those indicating hemolysis, icterus, or lipemia, were excluded. Serum samples were stored at −80°C to ensure stability until analysis. Serum postsurgical Tg lower than 1‐2 ng/mL is a strong predictor of remission, whereas Tg levels more than 10‐30 ng/mL are associated with persistent or recurrent disease.^19^, ^20^ We selected cases presenting different ranges of Tg concentrations (<0.2 ng/mL, 0.2‐50 ng/mL, and >50 ng/mL) measured by the two assays.

### 2.2 Assays

Tg was determined by using two different chemiluminescence immunoassays (CLIA). Specifically, these included Liaison (Liaison XL, Diasorin, Saluggia, Italy) and Beckman UniCeL DxI800 (Beckman Coulter Inc, Brea, CA, USA). TgAbs were determined by Siemens ADVIA Centaur (Siemens Healthcare Diagnostics Products Ltd., Glyn Rhonwy, Llanberis, Gwynedd, Wales, UK) system (reference cutoff = 60 UI/mL). All Tg assays were standardized 1:1 to the International Reference Preparation CRM‐457. The TgAbs assay was standardized to the WHO reference serum 65/93. The assays were performed according to the manufacturer's instructions. The quality control materials for the immune and tumor markers from the Bio‐Rad system (Bio‐Rad Laboratories Inc, Irvine, CA, USA) were used. Analytical measurement ranges (AMR) of serum Tg were from 0.1 to 500 ng/mL for both DxI 800 and Liaison XL. The analytical sensitivity (AS) limits for the Diasorin and Beckman systems were 0.1 ng/mL. The manufacturer‐recommended cutoffs for the Diasorin and Beckman systems were 54 and 50 ng/mL, respectively.

The Liaison Tg II confirmatory test was performed. We added confirmatory reagent to samples before Tg‐L measurement. Tg present in the confirmatory reagent binds antibodies anti‐Tg of the sample, and it will not be available for the antibodies used in the immunoassay. % Tg recovery was calculated by the following formula: [Tg concentration after confirmatory reagent addition divided by (0.8 × Tg concentration + 0.2 × Tg concentration of confirmatory reagent)] *100. The acceptable recovery range quoted by Diasorin Diagnostics is 70%‐130%. Tg is used also to assess response to therapy for well‐differentiated thyroid cancer: value of <0.2 ng/mL is consistent with an excellent response to therapy, whereas a biochemically incomplete response to therapy is a suppressed Tg >1 ng/mL or a stimulated Tg >10 ng/mL.^3^ Tg results obtained from the two CLIA systems below 0.2 ng/mL were considered negative for this study. The concordance rate refers to whether the results are evaluated as being in the same category according to different methods. For example, a positive coincidence rate indicates that the results are evaluated as positive by the two compared methods.

### 2.3 FNA‐Tg

After collection of FNA (performed on lymph node metastasis) cytology samples, each needle was washed with 1.0 mL of normal saline (0.9% w/v of NaCl) and sent to the laboratory. Blood contaminated FNAC needle washes were spun, and the clear supernatant was removed for thyroglobulin (Tg) testing. Blood‐free samples were tested without manipulation. In the needle washing, Tg‐L and Tg‐A (validated for use in cytology needle washouts collected using normal saline samples) were measured. A level higher than 1 ng/mL was considered positive.^10^ Residual FNA collected in our routine procedure was used.

### 2.4 Method comparison and statistical analysis

Serum Tg‐L and Tg‐A were compared by using 91 serum samples. The Pearson correlation coefficient (*r*) was calculated to determine the relationship between the results of the two assays. For serum postoperative Tg and FNA‐Tg, the agreement between results obtained by the two CLIA systems was evaluated as categorical variables divided by the cutoffs for positivity above indicated.^3^, ^10^ Bland‐Altman plot was used to assess bias.

## 3 RESULTS

### 3.1 Comparison of Tg‐L and Tg‐A for values ranging from 0.2 ng/mL to 50 ng/mL

**FIGURE 1:**
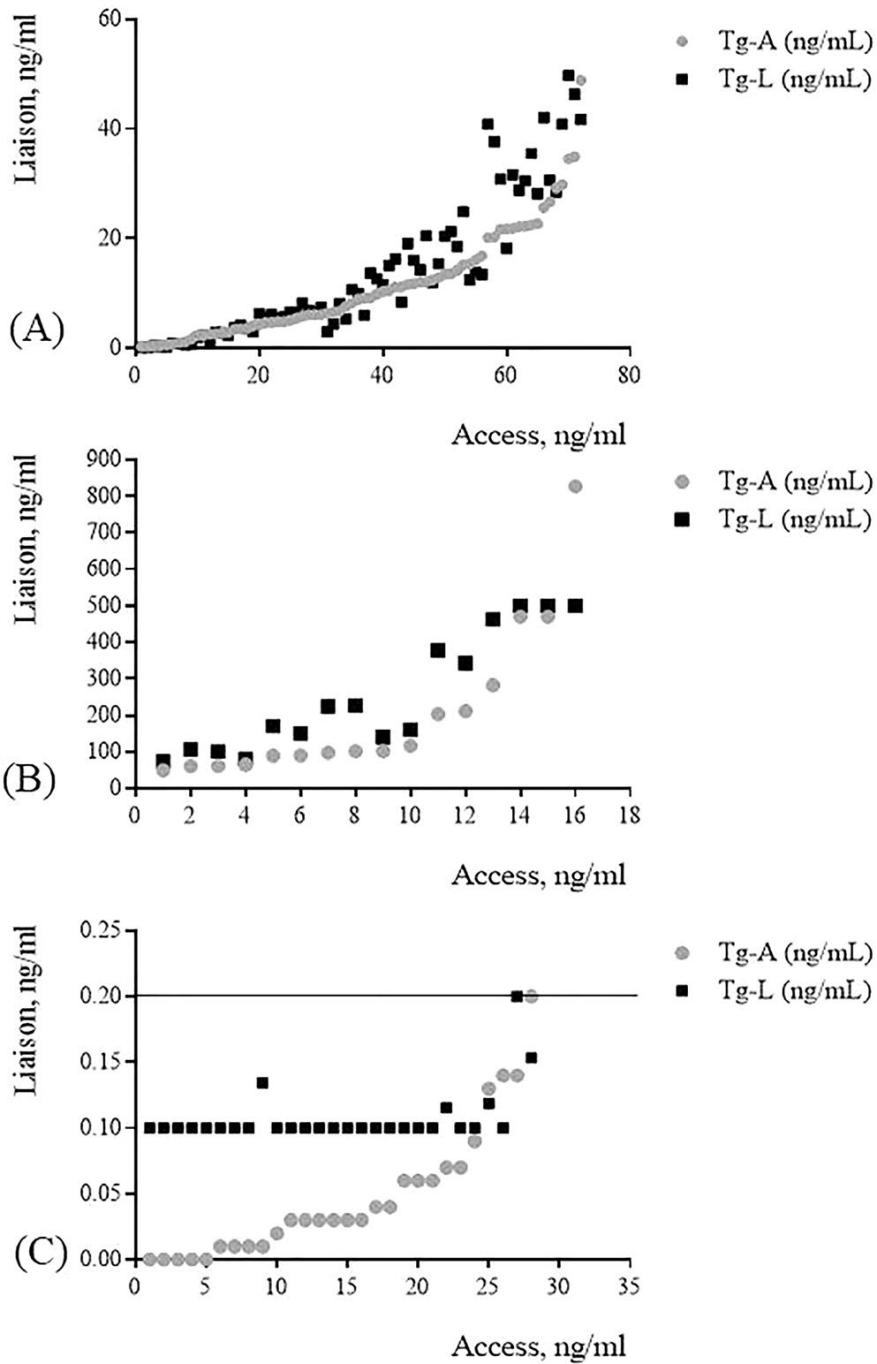
Pearson's correlation between the two CLIA systems. (A) Method correlation between Tg‐L and Tg‐A for Tg serum values ranging from 0.2 to 50 ng/mL; (B) Method correlation between Tg‐L and Tg‐A for Tg serum values higher than 50 ng/mL; (C) Method correlation between Tg‐L and Tg‐A for Tg serum values lower than 0.2 ng/mL

As shown in Figure 1A, a significant relationship between Tg‐L and Tg‐A assays was found (Pearson's *r* 0.933 [95%CI 0.894‐0.958). *P* < 0.001).

### 3.2 Comparison of Tg‐L and Tg‐A for values higher than 50 ng/mL

As shown in Figure 1B, a significant relationship between Tg‐L and Tg‐A assays was found (Pearson's *r* 0.849 [95%CI 0.609‐0.946 *P* < 0.001). However, there are significant variations in Tg values for concentrations higher than 200 ng/mL in the studied population and this may be a possible reason for large biases%.

### 3.3 Comparison of Tg‐L and Tg‐A for values lower than 0.2 ng/mL

The overall concordance rate in discriminating negative (<0.2 ng/mL) and positive (>0.2 ng/mL) Tg values was 92% (Figure 1C). In particular, 26/28 samples were negative in both assays. Among 2 discrepant results, 1 was 0.2 ng/mL in Liaison and undetectable in Access assay and the other viceversa. All these two patients have negative TgAbs values.

### 3.4 Comparability of Tg‐A and Tg‐L values

**FIGURE 2:**
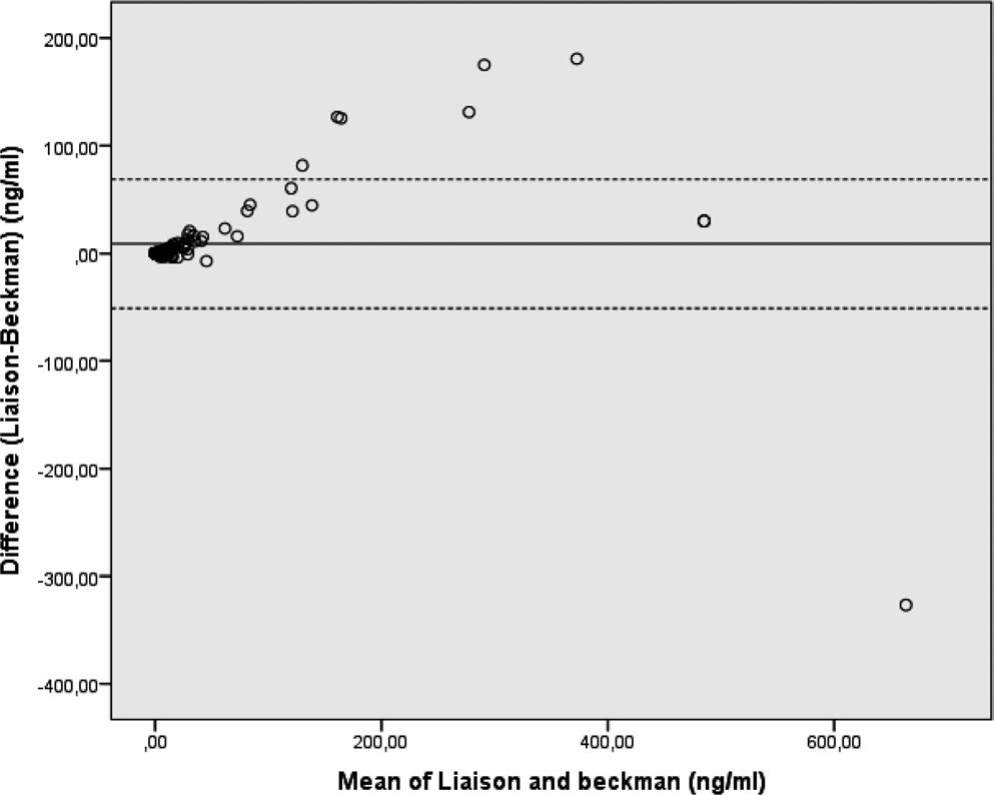
Bland‐Altman plot of Tg levels by Access and Liaison

Bland‐Altman plot (Figure 2) showed no significant differences among the two assays. The mean bias was 8.8 ng/mL (95%CI 0.54‐17.08, *P* = .037). The regression equation was Y = 0.009x + 8.452, and 95% CIs for the slope and intercept were 0.00016‐0.0183 and 8.144‐8.683, respectively.

### 3.5 Comparison of FNA‐Tg‐L and Tg‐A

We measured the value of Tg in fine‐needle aspiration biopsy washout fluid (FNA‐Tg) in 7 samples from DTC patients, using both CLIA systems. The overall concordance rate in discriminating negative (<1 ng/mL) and positive (>1 ng/mL) FNA‐Tg values was 100% (Table 1). In particular, 4 and 3 samples were positive and negative in both assays.

**TABLE 1:** Concordance between Tg‐L and Tg‐A in FNAs from 7 DTC patients

Patient	Tg‐A (ng/mL)	Tg‐ L (ng/mL)
1	<0.05	<0.1
2	<0.05	<0.1
3	0.18	0.1969
4	0.06	<0.1000
5	0.06	0.1278
6	39 471.96	>500
7	3439.59	>500

### 3.6 Comparison of Tg assay interference by TgAbs and RF

Of the 91 samples, 25% (23/91) were consistently measured as TgAbs positive and 75% (68/91) as TgAbs negative. Of note, for TgAb‐positive subjects, the linear relationship between Tg‐L and Tg‐A was good (Pearson's *r* 0.988 [95%CI 0.933‐1.021), *P* < 0.001). In particular, Liaison Tg II Gen confirmatory test showed that in serum sample of a patient with TgAb higher than 500 UI/mL, the addition of a solution containing human recombinant Tg (250 ng/mL) provides a recovery of 90.7% (Table 2). Moreover, we determined Tg values in a patient with non‐relapsing DTC and serum RF higher than 500 IU/mL and we found that Tg‐A was 88.8 ng/mL and Tg‐L was 0.18 ng/mL (Table 3).

**TABLE 2:** Tg‐L recovery after the addition of Liaison TgII confirmatory reagent in a patient with positive TgAbs

Tg‐L before (ng/mL)	Tg‐L after (ng/mL)	Recovery %	Diagnosis	TgAbs (IU/mL)
155.6	154.9	90.7	DTC	>500

**TABLE 3:** Concordance between Tg‐L and Tg‐A in a patient with not‐relapsing DTC and serum RF > 500 IU/mL

Tg‐A (ng/mL)	Tg‐L (ng/mL)
88.8	0.1815

## 4 DISCUSSION

Tg measurement is a relevant marker for follow‐up of patients with DTC.^21^ The evaluation of new Tg assay is crucial for a correct interpretation of serum Tg modifications during follow‐up.^21^ This preliminary study compared the newly introduced Liaison Tg II assay with the well‐established Access Tg assay in serum, including the possible influence of high levels of TgAb and RF. In addition, we performed a comparison of the two assays in FNA from nodules and lymph nodes of 7 DTC patients. Overall, Liaison and Access Tg assay showed a high correlation for Tg values ranging from 0.2 and 50 ng/mL and higher than 50 ng/mL. Interestingly, for serum Tg values lower than 0.2 ng/mL (corresponding to cutoff used for biochemical recurrence),^21^ we found that all the negative samples for Tg‐A were negative for Tg‐L. The correlation for serum Tg‐Land Tg‐A results for TgAb‐positive subjects was good. However, further studies on larger population are needed to assess whether the interference of TgAb levels with Tg measurement might be equivalent for the two CLIA systems. Conversely, the correlation for RF‐positive subject was poor. Finally, we demonstrated that FNA‐Tg results are totally consistent using the two CLIA systems. Collectively, our findings suggest that Liaison and Access Tg assays are highly correlated. In the evaluation of highly sensitive Tg assay, a hot topic is the potential for interference with Tg measurement. In fact, up to 30% of DTC patients were TgAb positive at the time of diagnosis.^22^ One of the most used approaches to reveal TgAbs interference is the recovery of added exogenous Tg. This is the approach of Liaison Tg II Gen confirmatory test. Confirmatory reactive can bind TgAbs in the sample. A comparison between Tg values before and after the addition of this reagent can indicate potential interference effects. Liaison Tg II Gen confirmatory test is clinically relevant, since even low TgAbs concentrations are able to potentially mask Tg epitopes used by reagent antibodies and put in the shade the very low Tg amount measured by highly sensitive assays.^23^ Investigation of the Liaison Tg II Gen confirmatory test showed that all samples, including those with known TgAb interference, had % Tg recovery within the acceptable recovery range quoted by Diasorin Diagnostics (70%‐130%). Confirmatory test did not identify patients with TgAb interference when the suggested acceptable recovery range was used. Further studies are required to determine if an alternative cutoff for recovery may improve the ability of this test in the identification of TgAb interference. This is a clinically relevant issue, since in a percentage of patients of about <10% interference with Tg measurement was found due to heterophile antibodies and RF.^16^, ^17^, ^24^, ^25^, ^26^ In patients with abnormal recovery test and/or Tg results discordant with clinical picture or by different analytical methods, an interference effect should be considered. Liquid chromatography‐tandem mass spectrometry (LC‐MS MS) is the only currently available methodology that could eliminate interference by TgAbs, heterophile antibodies, and RF.^7^, ^27^ However, LC‐MS MS has a manual workflow, is not widespread present in clinical laboratories, and does not present the necessary functional sensitivity.^28^ Therefore, the use of LC‐MS MS should be reserved for selected cases with TgAb positive and Tg undetectable, where Tg determination is essential for clinical‐decision making. In this context, Liaison Tg II Gen confirmatory test showed promising results in measuring Tg in biological samples with potential antibody interference using immunoassays. It is worthy of attention that Tg‐L seems to be not affected by RF interference, probably due to differences in the antibodies used as reagents in the two immunossays.^29^ However, we analyzed only one patient with high RF, so further studies on larger population will help to obtain a definitive conclusion. We also compared FNA‐Tg measurement by Liaison Tg II and Access assays. Several analytical factors such as lack of standards, variability of the commercially available antibody kits, and insufficient functional sensitivity may affect the accuracy of FNA‐Tg results, posing difficulties in the comparison of methods.^30^ Liaison Tg II and Access Tg assays have a very good functional sensitivity, giving consistent results also for Tg values ranging from 0.1 to 1 ng/mL. Moreover, both immunoassays are not based on electrochemiluminescence. Thus, they do not seem to be affected by matrix effects associated with the use of physiological saline.^31^ Collectively, Liaison Tg II and Access assays showed a good concordance of Tg measurement in FNA washout fluids (Table 3). Considering that Tg‐A is currently used for use in cytology needle washouts collected using normal saline samples, Tg‐L might be considered for this clinical use. In conclusion, our preliminary data on a small study population suggest a good correlation between Liaison Tg II assay and the well‐established Access immunoassay. Further studies on larger population are needed to confirm our findings and to evaluate the clinical performance of this new immunoassay in the follow‐up of DTC patients.

## AUTHOR CONTRIBUTIONS

All the authors have accepted responsibility for the entire content of this submitted manuscript and approved submission.
